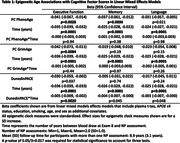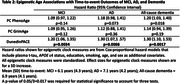# Epigenetic age acceleration and cognitive resilience in the Framingham Heart Study

**DOI:** 10.1002/alz.091707

**Published:** 2025-01-03

**Authors:** Ryan Dacey, Shruti Durape, Mengyao Wang, Phillip H Hwang, Ashita S. Gurnani, Ting Fang Alvin Ang, Sherral A. Devine, Seo‐Eun Choi, Michael L. Lee, Phoebe Scollard, Laura E. Gibbons, Shubhabrata Mukherjee, Emily H. Trittschuh, Richard Sherva, Logan C. Dumitrescu, Timothy J. Hohman, Michael L. Cuccaro, Andrew J. Saykin, Paul K. Crane, Yi Li, Daniel Levy, Jiantao Ma, Chunyu Liu, Kathryn L. Lunetta, Rhoda Au, Lindsay A. Farrer, Jesse Mez

**Affiliations:** ^1^ Department of Medicine (Biomedical Genetics), Boston University Chobanian & Avedisian School of Medicine, Boston, MA USA; ^2^ Framingham Heart Study, Boston University Chobanian & Avedisian School of Medicine, Boston, MA USA; ^3^ Boston University Alzheimer’s Disease Research Center, Boston University Chobanian & Avedisian School of Medicine, Boston, MA USA; ^4^ Department of Neurology, Boston University Chobanian & Avedisian School of Medicine, Boston, MA USA; ^5^ Department of Biostatistics, Boston University School of Public Health, Boston, MA USA; ^6^ Department of Epidemiology, Boston University School of Public Health, Boston, MA USA; ^7^ Slone Epidemiology Center, Boston University Chobanian & Avedisian School of Medicine, Boston, MA USA; ^8^ Department of Anatomy & Neurobiology, Boston University Chobanian & Avedisian School of Medicine, Boston, MA USA; ^9^ Department of Medicine, University of Washington School of Medicine, Seattle, WA USA; ^10^ University of Washington School of Medicine, Seattle, WA USA; ^11^ Geriatric Research, Education, and Clinical Center, Veterans Affairs Puget Sound Health Care System, Seattle, WA USA; ^12^ Department of Psychiatry and Behavioral Sciences, University of Washington School of Medicine, Seattle, WA USA; ^13^ Vanderbilt Memory and Alzheimer’s Center, Vanderbilt University Medical Center, Nashville, TN USA; ^14^ Vanderbilt Genetics Institute, Vanderbilt University Medical Center, Nashville, TN USA; ^15^ The John P. Hussman Institute for Human Genomics, University of Miami, Miami, FL USA; ^16^ Indiana Alzheimer’s Disease Research Center, Indiana University School of Medicine, Indianapolis, IN USA; ^17^ Indiana University School of Medicine, Indianapolis, IN USA; ^18^ Population Sciences Branch, Division of Intramural Research, National Heart, Lung, and Blood Institute, National Institutes of Health, Bethesda, MD USA; ^19^ Nutrition Epidemiology and Data Science, Friedman School of Nutrition Science and Policy, Tufts University, Boston, MA USA; ^20^ Boston University Chobanian & Avedisian School of Medicine and School of Public Health, Boston, MA USA; ^21^ Department of Ophthalmology, Boston University Chobanian & Avedisian School of Medicine, Boston, MA USA; ^22^ Framingham Heart Study, Boston, MA USA

## Abstract

**Background:**

There is growing evidence that epigenetic age acceleration may predict late life cognitive decline and dementia, but it is unknown whether this is due to accelerated neurodegeneration or reduction in cognitive resilience. We examined the relationship between epigenetic clocks and domain specific neuropsychological (NP) factor scores, mild cognitive impairment (MCI), Alzheimer’s Disease (AD), and all‐cause dementia, before and after accounting for plasma total tau (t‐tau), a marker of neurodegeneration.

**Method:**

DNA methylation and plasma t‐tau (Simoa assay; Quanterix) data from 2091 Framingham Heart Study Offspring cohort participants were generated from blood at the same Exam 8 visit (2005‐2008). Three epigenetic clock measures: DunedinPACE, PC PhenoAge, and PC GrimAge were estimated from the DNA methylation data. Longitudinal NP factor scores were previously derived for memory, language, and executive function using confirmatory factor analysis. We tested the association of epigenetic age acceleration with cognitive trajectories using linear mixed effects models and with time to MCI, all‐cause dementia and AD using Cox‐proportional hazard models. Models were run with and without adjustment for plasma t‐tau. All models included *APOE* ε4‐carrier status, education, smoking, age, and sex as covariates. Epigenetic measures were standardized in all models.

**Result:**

At Exam 8, the sample was, on average, 66.3 (SD = 9.0) years of age, 54.8% female, and had 16.4 (SD = 2.7) years of education. DundeinPACE was significantly associated with faster decline in executive function (β_timeXepi_age_ = ‐0.005, 95% CI:[‐0.009,‐0.002], p = 0.0020), but not with baseline executive function. Older PhenoAge (β_epi_age_ = ‐0.041, 95% CI:[‐0.067,‐0.014], p = 0.0028) and GrimAge (β_epi_age_ = ‐0.042, 95% CI:[‐0.073,‐0.011], p = 0.0084) were significantly associated with worse baseline executive function, but not with rate of decline. Older PhenoAge also was significantly associated with worse baseline memory (β_epi_age_ = ‐0.037, 95% CI:[‐0.061,‐0.012], p = 0.0036). DunedinPACE was significantly associated with time to MCI (HR = 1.20, 95% CI:[1.06,1.35], p = 0.0034), AD (HR = 1.30, 95% CI:[1.07,1.57], p = 0.0068) and all‐cause dementia (HR = 1.30, 95% CI:[1.10,1.53], p = 0.0017). Results remained similar after adjustment for plasma t‐tau.

**Conclusion:**

Epigenetic age acceleration may be a marker of cognitive resilience, particularly in executive function. Of the three epigenetic clocks examined, DundedinPACE showed the most robust associations with cognitive resilience, with lower DunedinPACE associated with greater cognitive resilience.